# A clinical trial of cognitive behavior therapy for psychiatric comorbidity and quality of life with Cancer Patients during Chemotherapy (CPdC)

**DOI:** 10.1186/s12888-022-03863-w

**Published:** 2022-03-29

**Authors:** Qasir Abbas, Nimra Arooj, Khawer Bilal Baig, Muhammad Umar Khan, Muhammad Khalid, Mafia Shahzadi

**Affiliations:** 1 Department of Applied Psychology, Government College University Faisalabad, Zakid Block 1st floor, Main Campus, Faisalabad, Punjab Pakistan; 2 Department of Professional Psychology, Bahria University Lahore Campus, Lahore, Pakistan; 3 Department of Clinical Oncology, Medical University Faisalabad, Faisalabad, Punjab Pakistan

## Abstract

**Background:**

Cancer is a common worldwide illness; it evokes psychological distress at different stages, during chemotherapy patient perceives a variety of psychiatric symptoms due to various medication side-effects and psychological distress. Studies have shown a significant impact of cognitive behavior therapy (CBT) in the management of psychiatric symptoms during chemotherapy. This study aims to investigate the effectiveness of CBT for depression, anxiety, stress, death anxiety, satisfaction with life, and self-esteem among cancer patients during chemotherapy (CPdC).

**Methods:**

Place and duration of the study: Department of Applied Psychology, Government College University Faisalabad in collaboration with Department of Oncology, Allied Hospital Faisalabad from November 20, 2020 and July 31, 2021. A total of 90 cancer patients were enrolled. 70 out of 90 met the eligibility criteria and 60 participants fulfilled all requirements. Participants were randomly allocated to four different groups. The pre-assessment screening was started along with the first trial of chemotherapy. The CBT-based treatment plan was formulated and one session per week was given to each patient for 3 to 4 months. Participants’ age range was 18–65 years (M ± SD = 47.51 ± 12.36. Demographic form, Depression Anxiety and Stress Scale (DASS), Death Anxiety Scale (DAS), Satisfaction with Life Scale (SWLS), and Rosenberg Self-Esteem Scale (RSES) were administered. Descriptive, t-test, and repeated measure ANOVA statistics were used to investigate the findings.

**Results:**

Results indicated significant mean difference on the variable of depression, anxiety and stress across four conditions (i.e. F(2, 56) = 39.55, *p* < .000, η^2^ = .679; F(2,56) = 73.32, *p* < .000, η^2^ = .797; F(2,56) = 119.77, *p* < .000, η^2^ = .865 respectively). On death anxiety significant difference across four conditions was found (F(2,56) = 22.71, *p* < .000, η^2^ = .549) with large effect size. Furthermore, findings indicated significant mean difference on the variable of satisfaction with life and self-esteem across four conditions was found (F(2,56) = 22.05, *p* < .000, η^2^ = .542; F(2,56) = 36.19, *p* < .000, η^2^ = .660) with large effect size.

**Conclusion:**

It is concluded that CBT played a very effective role to reduce depression, anxiety, and stress-related psychiatric symptoms. CBT reduces the level of death anxiety and improving the quality of life and level of self-esteem among CPdC.

**Trial Registration:**

The study trial was registered in the Thai Clinical Trial Registry-TCTR (TCTR20201113002).

## Introduction

Cancer is a life-threatening disease and a major leading cause of death globally. The Cancer mortality rate is increasing, approximately7.6 million deaths happened in 2008, and this mortality rate reached 19.3 million in 2020 [1]. In Africa, Asia, Central, and South America, more than 60% of cases and 70% of deaths are reported because of cancer (WHO, 2012). In 2020, a recent estimate said 0.18 million new cases were reported in Pakistan, and approximately 0.12 million deaths happened out of 220 million population (International Agency for Research on Cancer, 2020). In Pakistan, the breast cancer rate is estimated at 31.5%, comparatively 2.5% higher than in Iran and India [2]. In Pakistan, women are at high risk of breast cancer and men with prostate cancer [3]. Around 50% of women ignore their physical health status, do not get proper health examinations timely, and die due to increased severity (Public Health, 2014).

Cancer comorbidity is common [4] with depressive disorder [5], sleep disorders [6], anxiety-related disorder [7], and other psychiatric disorders [8]. Psychiatric symptoms (i.e., anxiety, fear, worries, restlessness, fatigue, low mood, sleep, and appetite problems) are reported higher in CPdC [9]. Studies have proven that psychological treatment effectively treats emotional and psychiatric problems among CPdC [10]. Cognitive behavior therapy (CBT) is a more promising approach to treat psychiatric problems and emotional vulnerabilities of CPdC [11]. CBT interventions address enormously depressive symptoms in cancer patients during chemotherapy [12]. CBT work starts from psychoeducation and continues until recovery [13]. Further, CBT creates an insight, addresses cognitive and behavioral problems, and provides skills training to CPdC [14].

Studies on the cancer patients related to their psychiatric problems are already rare. No one study is conducted in Pakistan on cancer patients to treat their psychiatric problems, particularly during chemotherapy. The general aim of this study was to investigate the impact of CBT on treating mental health problems, to improve patients’ quality of life and adherence to treatment.

## Methodology

### Research design

This clinical trial was approved by the Institutional Review Board (IBR), Government College University Faisalabad (Ref.No.GCUF/ERC/1996). Later on, the study protocol was approved by the Thai Clinical Trial Registry-TCTR on November 13, 2020 (TCTR20201113002). After approval from the registry, participants’ enrollment period was from November 20, 2020, to July 31, 2021. In this study, all methods were performed in accordance with the relevant guidelines and regulations. Written informed consent was obtained from all the participants. All patients read the consent form and signed it before enrolling in the study.

This experimental study was done using between group research design. After completing all the codal formalities, the, patients were allocated to each group i.e. experimental =35 (stage-I = 17 & stage-II = 18) and waitlist control =35 (Stage-I = 17 & Stage-II = 18) at baselines assessment and at follow up phase, after further attrition, the number of participants in experimental group was 30 (i.e. stage-I = 15 & stage-II = 15) and in waitlist control was also 30 (i.e. stage-I = 15 & stage-II = 15).

**Fig. 1:**
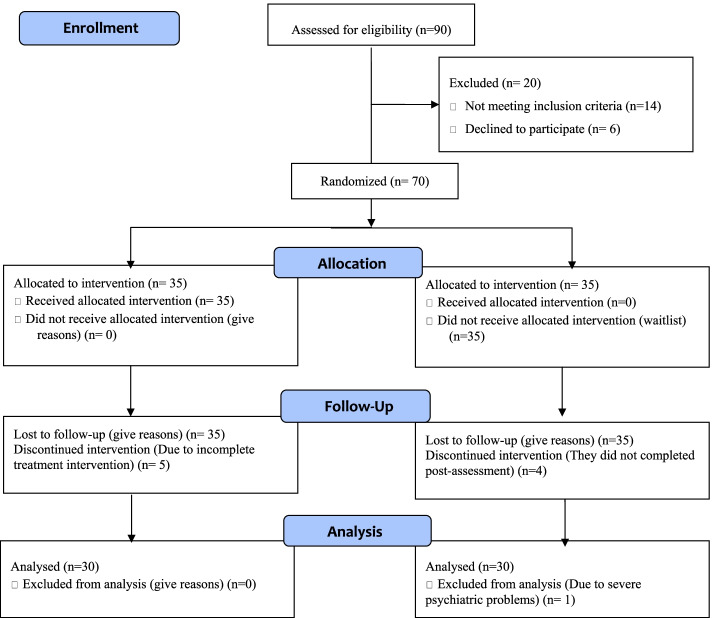
Flow diagram of Cancer Patients during Chemotherapy (CPdC)

The study recruited 90 cancer patients from the Department of Clinical Oncology, Allied Hospital Faisalabad, between November 2020 and July 2021. 70 out of 90 participants met the inclusion and exclusion criteria. Participants’ attrition rate was 67.78%, and the rest of the 32.23% was excluded. For example, patients who refused to participate in the study were 11.12%, excluded due to comorbidity problems were 6.67%, patients moved to other hospitals were 2.23%, shifted to different cities were 2.23%, missed various follow-up sessions were 5.56%, and not participated in post-assessment were 4.45% (see Fig. 1). Participants’ age range was 25–65 years (M = 47.51, SD ± 12.36). However, as the study progressed and the originally small differences present in terms of gender-ratio, and marital status grew large when many participants left the study (more than 30). Albeit, this attrition of participants that resulted in seemingly disproportionate experimental and control groups was not a threat to internal validity as all other relevant measured variables of concern were balanced in both groups beforehand. Our study has achieved variance minimization in the desired measured variables as opposed to pure randomization across all measured variables which is in line with the recommendations of Sella et al. [15].

#### Inclusion and exclusion criteria

All the patients were screened and diagnosed by the consultant oncologist. Only cancer patients of stages I and II were included in this study. Participants with other medical comorbidities were excluded from the study. Participants with any apparaently severe physicalinjury, amputation, and/or loss of autonomous mobilitywere not listed in the study. Furthermore, participants with a history of psychotic illness, or other psychiatric disorders were excluded. Additionally, people who had difficulty understanding and reciprocating verbal communication (due to any reason) were also excluded from the study. Those patients who initially met the inclusion criteria but later faced significant health decline were also excluded.

### Instruments

Demographic form and history-taking form were used for personal information and history of the problem. Then Urdu version Depression Anxiety and Stress Scale (DASS), a 42 item self-report measure was used [16, 17]. The DASS is a 4-point Likert scale with a response category ranging from “did not apply to me all=0” to “applies to me most of the time=3”. Further, DASS provides ranges of severity from *normal* to *extremely severe*. The internal consistency of depression is 0.91, anxiety is 0.84, and stress is 0.90. Urdu version of the Satisfaction with Life Scale (SWLS) was used [18, 19]. SWLS is a 7 point Likert scale. Each item is scored from “strongly agree =7” to “strongly disagree = 1”. SWLS describes ranges from extremely satisfied to highly dissatisfied. SWLS has a sound internal consistency of 0.74 (Cronbach’s alpha). The Death Anxiety Scale (DAS) developed by Templer [20] and adapted by Saleem et al. [21]. The DAS investigates the level of death anxiety among cancer patients. Each statement is scored with a “yes” or “no” response. A score range of 9–5 indicates-high a level of death anxiety, and a range of 4–8 indicates a medium level. Scale test-retest reliability coefficient is 0.83, with an internal consistency coefficient of 0.76. In addition, an Urdu version of the Rosenberg Self-Esteem Scale (RSES) was used [22, 23]. RSES consists of 10 items which are 4 points Likert scale from “strongly agree = 3” to “strongly disagree = 0”. High scores indicate a high level of self-esteem. The scale has a good reliability estimation of 0.87.

### Intervention

CBT is an evidence-based approach to treat psychological problems of cancer patients [24, 25]. Structured treatment plan was formulated with set agendas, and all goals were achieved in therapeutic sessions. The 1st agenda was to “*psycho*-*educate”* the patients about the nature of their problem. Through 2nd agenda *cognitive restructuring,* they were educated on how they can address their cognitive errors and negative beliefs (which are causing an emotional disturbance). The 3rd agenda was to teach the patient about *“identification of thoughts and core beliefs”* and how patients can identify, evaluate, and respond to automatic thoughts and core beliefs. The 4th agenda was *“stress and cancer crisis management”*. The therapist trained the patients using various techniques to address stressors and crisis interventions [26]. The 5th agenda based on *“motivation/boosters”* to sustain during and after treatment to earn positive outcomes and hopes. The 6th agenda is based on “problem-solving skills”, cancer patients usually find themselves unable to manage stress, so it is important to strengthen their positive ways of managing stress [27]. The 7th agenda was to engage the patients in *“leisure activities”* to maintain a pleasant environment, pleasure activities, family gathering, and frequent social involvement [28]. The 8th agenda of the therapy was to boost up patients’ *“coping strategies”* to overcome the psychological vulnerabilities. The 9th agenda involved the patients in therapy on regular bases performing various regular *“exercises and homework assignments”* to process therapy outcomes faster. The 10th agenda of the therapy was *“lapse-relapse prevention”.*

### Statistical analysis

Data was scrutinized and prepared for statistical analysis. Descriptive statistics (mean, standard deviation & frequency) were calculated to distribute and make the group comparable at the time of baseline assessment. Furthermore, Chi-square test for categorical variables and independent samples t-test, were used to compare the means between experimental and waitlist control on assessment variables. The *P* > .05 was used to test the variables that they are comparable and have no differences. To check the normality of the distribution (experimental and control group combined), skewness and kurtosis were used to check the sample normal distribution and S-K values were between +1.96 to – 1.96 which indicates that the sample was normally distributed. Furthermore, repeated measure ANOVA test was used to find out the differences between experimental and control group. An alpha of .05 was used to perform all analyses with *p-*value <.05 using IBM SPSS Statistics (Version 24).

## Results

There were no significant differences in pre-trearment measures (baseline assessment) in demographics characteristics and psychological measures between experimental and control groups (age 44.85 ± 10.87 vs. 45.60 ± 13.55 years, total age 47.73 ± 12.53 years, *p* = . > .05; age at diagnosis 43.99 ± 10.68 vs. 44.73 ± 12.9, total 46.86 ± 12.10 years *p* = . > .05; years of schooling 10.80 ± 2.29 vs. 10.29 ± 2.38, total 10.54 ± 2.33 years *p* = . > .05; depression 20.12 ± 5.0 vs. 18.34 ± 5.24, *p* = . > .05; anxiety 19.94 ± 6.38 vs. 16.14 ± 5.42, *p* = . > .05; stress 24.97 ± 6.17 vs. 21.94 ± 6.51, *p* = . > .05; death anxiety 11.20 ± 3.22 vs. 10.40 ± 3.19, *p* = . > .05; satisfaction with life 13.37 ± 13.52 vs. 13.52 ± 2.97, *p* = . > .05; self-esteem 14.52 ± 3.49 vs. 16.94 ± 5.32, *p* = . > .05) respectively (Table 1).

**Table 1:** Comparison of participants’ demographic characteristics groups wise and overall

Group				
Characteristics		Experimental	Waitlist Control	Total
		M (SD)	M (SD)	M (SD)
Age		44.85 (10.87)	45.60 (13.55)	47.73 (12.53)
Age at diagnosis		43.99 (10.68)	44.73 (12.90)	46.86 (12.10)
Gender M/F (n/%)		9 (25.7)/26 (74.3)	24 (86.6)/11 (31.4)	33 (47.1)/37 (52.9)
Marital Status S/M (n/%)		9 (25.7)/26 (74.3)	18 (51.4)/17 (48.6)	27 (38.6)/43 (61.4)
Monthly income		22,142.86 (17,148.39)	27,342.86 (19,060.19)	24,742.86 (18,187.18)
Residence Rural/Urban%		12 (34.3)/23 (65.7)	10,928.6)/25 (71.4)	22 (31.4)/48 (68.6)
Education	Below Metric (n/%)	09 (25.7)	15 (42.9)	24 (34.3)
	High school (n/%)	12 (34.3)	07 (20.0)	19 (27.1)
	Intermediate (n/%)	05 (14.3)	06 (17.1)	11 (15.7)
	Graduation & above (n/%)	09 (25.7)	07 (20.0)	16 (22.9)
	Overall Years of Schooling	10.80 (2.29)	10.29 (2.38)	10.54 (2.33)
Occupation	Currently working (n/%)	13 (37.1)	11 (31.4)	25 (35.7)
	Not working (n/%)	10 (28.6)	12 (34.3)	27 (38.6)
	Dependent on family (n/%)	12 (34.3)	12 (34.3)	18 (25.7)
Types of cancer	Brest cancer	18 (51.4)	10 (28.6)	28 (40.0)
	Lungs cancer	10 (28.6)	01 (2.90)	11 (17.5)
	Leukemia	04 (11.4)	14 (40.0)	18 (25.7)
	Carcinoma	03 (08.6)	10 (28.6)	13 (18.6)
Cancer Stages	Stage-I	17	17	–
	Stage-II	18	18	–
	Total	35	35	70
Depression scale		20.12 (5.00)	18.34 (5.24)	19.23 (5.17)
Anxiety scale		19.94 (6.38)	16.14 (5.42)	18.04 (6.18)
Stress scale		24.97 (6.17)	21.94 (6.51)	23.46 (6.48)
Death anxiety scale		11.20 (3.22)	10.40 (3.19)	10.80 (3.21)
Satisfaction with life scale		13.37 (2.68)	13.52 (2.97)	13.44 (2.81)
Self-esteem scale		14.52 (3.49)	16.94 (5.32)	15.73 (4.63)

*M/F* Males/Females, *S/M* Single/Married; *M* Mean, *SD* Standard Deviation

Results (Table 2) indicated significant mean difference on the variable of depression, anxiety and stress across four conditions (i.e. F (2, 56) = 39.55, *p* < .000, *η*^2^ = .679; F (2,56) = 73.32, *p* < .000, *η*^2^ = .797; F(2,56) = 119.77, *p* < .000, *η*^2^ = .865 respectively) with large effect size. Results reveal that depression, anxiety, and stress-related symptoms significantly reduced after CBT treatment in stage-I (M ± SD = 5.0 ± 2.91; 5.07 ± 2.97; 6.86 ± 2.18) and stage-II (M ± SD = 6.25 ± 2.29; 4.93 ± 2.91; 8.50 ± 2.25) as compared to the control group in stage-I (M ± SD = 18.40 ± 6.95; 16.67 ± 6.0; 22.20 ± 6.76) and stage-II (M ± SD = 17.74 ± 7.23; 15.34 ± 6.16; 22.47 ± 6.17). The paired wise comparisons indicated significant means differences between experimental and control groups on depression, anxiety, and stress-related symptoms. On death anxiety significant difference across four conditions was found (F (2,56) = 22.71, *p* < .000, *η*^2^ = .549) with large effect size. Result reveals that death anxiety significantly reduced after CBT treatment in stage-I (M ± SD = 3.14 ± 0.87) and stage-II (M ± SD = 4.38 ± 1.93) as compared to the control group in stage-I (M ± SD = 11.00 ± 2.83) and stage-II (M ± SD = 9.34 ± 2.56). The paired wise comparisons indicated significant means differences between experimental and control group on death anxiety. Furthermore, findings indicated significant mean difference on the variable of satisfaction with life and self-esteem across four conditions was found (F(2,56) = 22.05, *p* < .000, *η*^2^ = .542; F(2,56) = 36.19, *p* < .000, *η*^2^ = .660) with large effect size. Results reveal that satisfaction with life and self-esteem significantly improved after CBT treatment in stage-I (M ± SD = 22.15 ± 3.66; 20.0 ± 2.26) and stage-II (M ± SD = 24.06 ± 5.63; 22.31 ± 1.86) as compared to the control group in stage-I (M ± SD = 13.93 ± 2.66; 15.07 ± 2.80) and stage-II (M ± SD = 14.20 ± 3.43; 17.27 ± 4.84). The paired wise comparisons indicated significant means differences between experimental and control groups on the variable of satisfaction with life and self-esteem. Overall, results explain that experimental group was found significant different from control group.

**Table 2:** Mean (standard deviation) and repeated measure ANOVA was used to investigate the difference between pre- and post-assessment results

	Groups									
	Experimental				Waitlist Control					
	Stage-I		Stage-II		Stage-I		Stage-II			
	Baseline M (SD)	Post-Test M (SD)	Baseline M (SD)	Post-Test M (SD)	Baseline M (SD)	Post-Test M (SD)	Baseline M (SD)	Post-Test M (SD)	F	*η*_p_^2^
DEPS	23.29 (3.87)	5.00 (2.91)	19.25 (4.10)	6.25 (2.29)	18.98 (5.24)	18.40 (6.95)	16.93 (5.22)	17.74 (7.23)	39.55***	.679
ANXS	21.72 (5.61)	5.07 (2.97)	20.75 (6.06)	4.93 (2.91)	15.87 (5.36)	16.67 (6.00)	14.34 (6.44)	15.34 (6.16)	73.32***	.797
STSS	29.21 (4.12)	6.86 (2.18)	22.32 (6.11)	8.50 (2.25)	21.13 (6.36)	22.20 (6.76)	22.14 (6.20)	22.47 (6.17)	119.77***	.865
DAS	11.85 (1.61)	3.14 (0.87)	11.06 (4.24)	4.38 (1.93)	11.00 (2.83)	9.67 (2.36)	9.74 (3.22)	9.34 (2.56)	22.71***	.549
SWLS	12.36 (2.93)	22.15 (3.66)	14.40 (2.64)	24.06 (5.63)	13.47 (3.14)	13.93 (2.66)	13.60 (2.83)	14.20 (3.43)	22.05***	.542
RSES	13.57 (3.44)	20.00 (2.26)	15.63 (3.85)	22.13 (1.86)	15.40 (2.83)	15.07 (2.83)	16.87 (5.72)	17.27 (4.84)	36.19***	.660

*** = *p* < .001; η_p_^2^; Partial Squared Eta; *DEPS* Depression Scale, *ANXS* Anxiety Scale, *STSS* Stress Scale, *DAS* Death Anxiety Scale, *SWLS* Satisfaction with Life Scale, *RSES* Rosenber Self-Esteem Scale, *M* Mean, *SD* Standard Deviation

**Fig. 2:**
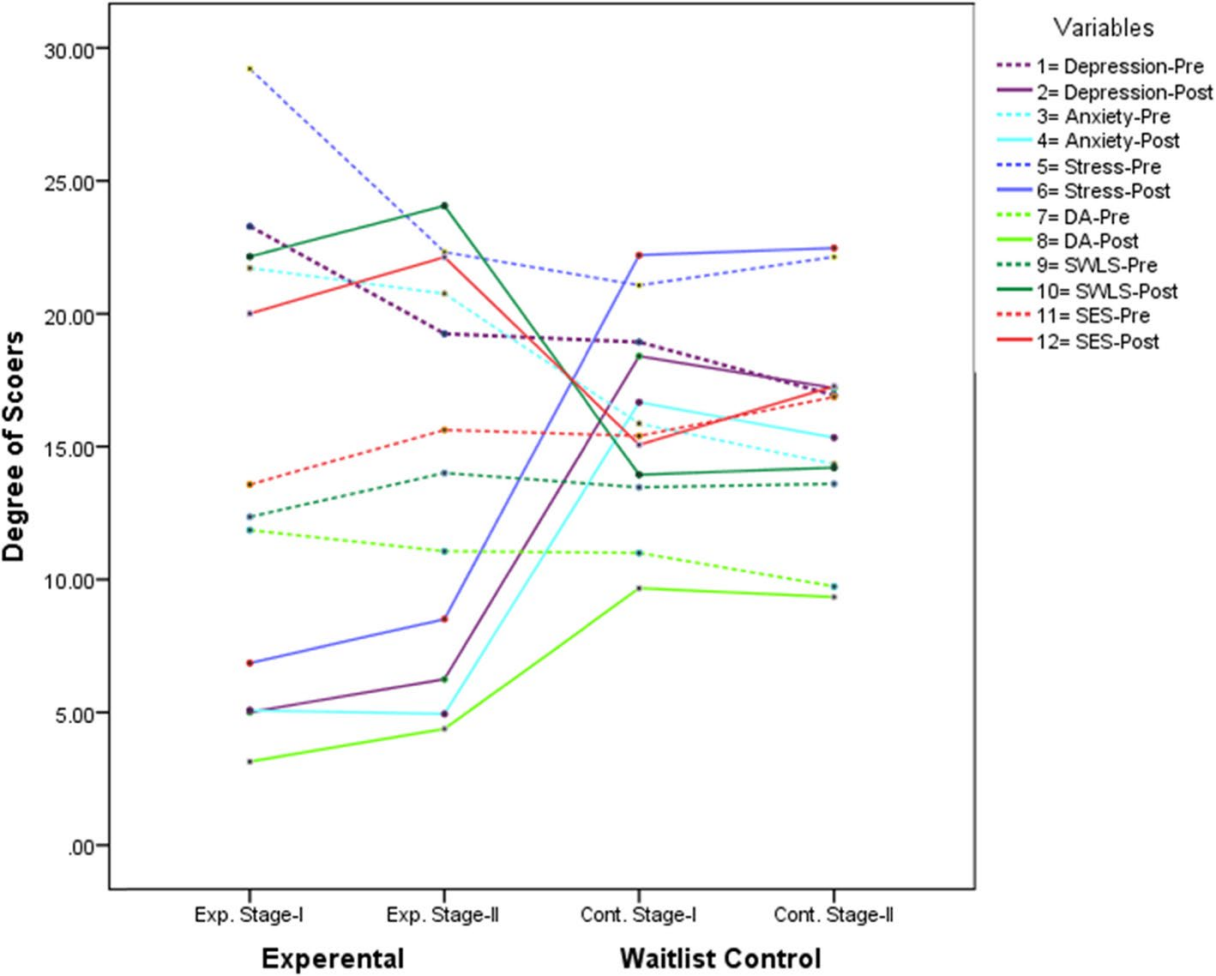
Pre- and post-assessment analysis on the clinical variables between experimental and control groups

Graphical presentation of the results reveals that CBT produced substantial improvement on post-testing scores in the experimental group of stage-I and II. Similarly, participants with stage-I and II significantly reduced the level of depression, anxiety, stress, and death anxiety, and significantly improved the level of satisfaction with life and self-esteem scale as compared to the control group of stage-I and II among CPdC (see Fig. 2).

## Discussion

Overall findings appear to be in the same direction as those found in the literature regarding the high efficacy of CBT in treating cancer patients during chemotherapy to manage psychological symptoms [28]. CPdC experienced illness stress which leads to anxiety and depressive symptoms. It was appraised that CBT positively addresses depressive symptoms of low mood, lack of interest, sleeping, and appetite problems in cancer patients. Similarly, patients with cancer stage I and II from the experimental group were found not significantly different, but they were found significantly different from patients with cancer stage I and II of a control group. Furthermore, overall it was found that there were no significant differences across the stages of cancer within both experimental and control groups on all the measured variables; this indicates that the stage of cancer has did not make any significant difference for cancer patients. Herein, CBT interventions reduced the severity of overall clinical symptoms and increased patients’ willpower and confidence to face the illness’ adverse effects and restructure thoughts regardless of the stage of the cancer [29].

In therapy, our emphasis was to train the patients in terms of “*psychoeducation*, *identification of thoughts and core beliefs, cognitive restructuring, inoculation of motivational thoughts/beliefs,* and development of new skills; *stress and cancer crisis management, problem-solving, coping strategies, leisure activities”* [30]*.* Studies have shown a positive impact of CBT on addressing daily life stressors, identifying cognitive errors and thought restructuring, coping skills, and leisure activities [31]. When a therapist provided psychoeducation and awareness to patients about the nature of the illness, patients started to overcome the emotional problems through coping mechanisms [32]. The outcomes were evidence of a decrease in the level of psychological distress and increased willpower with high motivations, mood stability, and the ability to cope with stress throughout treatment [33].

The cognitive intervention proved beneficial to minimize anxiety symptoms. The CPdC patients, who received CBT interventions, exhibited an ability to manage death-related anxiety [34]. The therapist properly educated the patients using different techniques and skill training; they reduced fear of death anxiety, and psychological distress. It was observed CBT interventions reduced the intensity of anxiety disorders at the early stage of diagnosis as well as the early detection of anxiety-related symptoms, while the undetected symptoms will lead to severe anxiety disorders in later life [24].

CBT treatment significantly improved the level of life satisfaction and self-esteem among CPdC. During chemotherapy, patients usually experience somatic complaints and adverse side effects, which elevate patients’ mood swings cause dissatisfaction with life [13] and other associated extraneous factors increase more negative outcomes [35]. Further, individuals with low self-esteem perceived high scores on depression and low scores on life satisfaction during cancer treatment because of less responsible, lower need for achievement, and poor motivation, while patients with high scores on self-esteem perceived a low degree of depression and anxiety because theyare more stronger to control emotions and encourgment to bear the pain during treatment [12]. Thus, CBT focuses on developing illness acceptance and self-management to improve treatment efficacy and lower psychological disturbance [36]. CBT played a supporting role along with the chemotherapy trial to manage and overcome psychiatric symptoms; psychological distress was reduced, and there were improvements in the patient’s quality of life [37]. To our knowledge, it was the first CBT trial in Pakistan that was applied to cancer patients during chemotherapy.

## Conclusion

In conclusion, we found CBT an evidence-based intervention with statistically significant efficacy and feasibility to treat psychiatric symptoms, reduce fears/apprehensions, improve mood stability, self-image, and satisfaction with life. CBT efficiently addresses maladaptive schemas and beliefs they trigger due to emotional disturbance and affects physical and mental health during chemotherapy.

### Limitations and recommendations

This study was the first initiative in Pakistan to develop an evidence-based treatment model of CBT with cancer patients during chemotherapy. This is recommended along with chemotherapy as an additional and supportive treatment to produce better outcomes. Further study should find its efficacy with cancer patients at all stages, focusing on other psychological problems except depression and anxiety. Patients were not familiar with psychological interventions; therefore, the termination rate was high, and some patients did not practice these interventions properly due to a lack of awareness about their efficacy.

### Clinical implications

This study provides a rationale background to practitioners that they can apply psychological interventions as supportive treatment modalities along with chemotherapy, increasing treatment adherence of the cancer patients. Psychoeducation, awareness, and psychological interventions are the most relevant treatment strategies that significantly address psychological problems and improve patients’ willpower and support of chemotherapy to produce better outcomes.

## Data Availability

The dataset generated and/or analyzed during the present study are not publically available because no permission was taken from the participants and the hospital administration where the study was conducted. The datasets are available from the corresponding authors on a reasonable request.

## Abbreviations

CBT: Cognitive behavior therapy
CPdC: Cancer patients during chemotherapy
DASS: Depression anxiety stress scale
DAS: Death anxiety scale
SwLS: Satisfaction with life scale
RSES: Rosenberg self-esteem scale
ANOVA: Analysis of variance
TCTR: Thai clinical trial registration
WHO: World health organization
IBR: Institutional review board
M/F: Male/female
M: Mean
SD: Standard deviation
DEPS: Depression scale
ANXS: Anxiety scale
STSS: Stress scale
